# Just One Shot? The Contextual Effects of Matched and Unmatched Intoxication on Perceptions of Consent in Ambiguous Alcohol-fueled Sexual Encounters

**DOI:** 10.1177/08862605231182378

**Published:** 2023-07-10

**Authors:** Ellen Laughlin, Molly Pettitt, Veronica M. Lamarche, Laurie James-Hawkins

**Affiliations:** 1 University of Essex, Colchester, UK

**Keywords:** alcohol, consent, sexual assault, gender, sexual minority orientations

## Abstract

The current research examined how contextual factors—the quantity of alcohol consumed by each partner, and whether this quantity matched—influenced how alcohol-fueled sexual encounters were perceived with regard to consent, coercion, sexual assault, and perceived responsibility of the focal partner for the outcome of the encounter. Across four studies (*N*_total_ = 535), participants read vignettes in which one person described a sexual encounter they had following a night out drinking. These scenarios differed within studies as a function of quantified alcohol consumed (1 shot; 15 shots) and whether both people in the vignettes consumed the same amount of alcohol (matched; unmatched). They also differed between studies as a function of whether the couples described were mixed gender or same gender. Across all four studies, scenarios in which both people in the scenario consumed different quantities of alcohol (i.e., 15 vs. 1 shot) were seen as less consensual, more coercive, and more likely to be an assault compared to scenarios where consumption was matched, especially at lower levels of intoxication (i.e., 1 shot each vs. 15 shots each). However, focal partners were also seen as *less* responsible for the outcome of the interaction when levels of intoxication were unmatched compared to matched. This pattern replicated across scenarios depicting same-gender and mixed-gender couples. These findings suggest that people prioritize information regarding whether sexual partners are “matched” or “unmatched” in terms of their intoxication when evaluating whether ambiguous sexual encounters are consensual and perceived individual responsibility.

Sexual victimization continues to be a pervasive social issue globally, across both developing (Jewkes et al., 2005; Simon-Kumar, 2014) and industrialized countries (Brennan & Taylor-Butts, 2008; Waterhouse et al., 2016). It is estimated that 1 in 5 women and 1 in 71 men will experience sexual assault at some point in their lives (Black et al., 2010). Incidences of sexual victimization are even more prevalent among marginalized populations, with approximately 44% of lesbians, 61% of bisexual women, 26% gay men, and 37% bisexual men experiencing rape, physical violence, or stalking by an intimate partner (The Centers for Disease Control and Prevention, 2010). These estimates likely understate the scope of the problem, as many sexual assaults go unreported.

Traditional rape scripts often mislead people into falsely believing that most sexual assaults are random violent attacks perpetrated by opportunistic criminals (Anderson, 2007). In reality, an estimated four in five sexual assaults happen in contexts where consensual sex could have been a possibility (e.g., at a party; Banyard et al., 2007). Furthermore, most perpetrators are not strangers, but typically someone known to victims, such as a friend or romantic partner (Waterhouse et al., 2016). Alcohol also plays a role in sexual assaults. Approximately 50% of reported sexual assaults have occurred where either the perpetrator and/or the victim were consuming alcohol (Abbey et al., 1996). Alcohol consumption is therefore an important contextual factor that has been shown to influence perceptions of potential partner sexual receptivity (Bogren et al., 2023; Farris et al., 2010; Hunt et al., 2021; Romero-Sánchez et al., 2012). Information about alcohol consumption can also influence how impartial observers perceive the encounter and evaluate consent.

However, most research exploring the impact of alcohol consumption before a sexual encounter treats intoxication as an abstract concept, typically mentioning that one or both people were “drunk” but without quantifying what that may mean. This does not match the lived experiences of young people today and may therefore contribute to the perceived ambiguity of the context in which the sexual encounter occurred. For instance, many young adults keep a relatively accurate count of their alcohol consumption (Northcote & Livingston, 2011) and often have specific strategies for reaching a target level of drunkenness (Zajdow & MacLean, 2014). Intoxication is therefore not a binary, but rather a state that can increase incrementally. This has important implications for understanding how people may interpret what it means to have sex when “drunk.” Thus, referring to one or more partners who engaged in sexual acts as “drunk” may contribute to contextual ambiguity (e.g., were they buzzed vs. drunk but still aware of their surroundings vs. “black out” drunk), leaving more room for interpretation for how behaviors and decision-making were likely impaired, when compared to concrete information that calibrates exactly how impaired someone would likely be.

The impact of alcohol on perceptions of sexual interactions is further influenced by legal definitions of consent that may or may not explicitly reference intoxication. In the United Kingdom (UK), for example, section 74 of the Sexual Offences Act 2003 states that an individual is only unable to consent to sexual interactions if they lack the freedom and capacity to do so. For example, being under the legal age of consent or having a mental disorder means that the individual lacks capacity to consent to sexual interactions. However, the legality surrounding alcohol consumption and intoxication leaves more room for interpretation by the people deciding whether to engage in sex. UK law regarding alcohol and consent only states that a person should not be incapacitated by drink and advises that the capacity to consent may diminish before they are unconscious (e.g., Crown Prosecution Service, 2021). Following this logic, although it may still be morally wrong to have sex with an intoxicated partner, it is not always illegal (Clough, 2019). Thus, simply being “drunk” or “very drunk” does not unambiguously mean that an individual could not legally give consent. Indeed, both partners being equally intoxicated can change how people evaluate sexual encounters (Hunt et al., 2021; James-Hawkins & Lamarche, forthcoming). For example, people fell into two different categories when evaluating whether hypothetical hookups described a sexual assault: (a) those who believed both people being equally intoxicated represented an assault because it represented diminished capacity (i.e., consistent with the legal definition), and (b) those who believed both people being intoxicated negated the culpability of any one individual (i.e., two wrongs make it right) (James-Hawkins & Lamarche, forthcoming). Thus, both quantity of alcohol consumed, as well as the relatively “matched” intoxication level of sexual partners influences how people perceive sexual encounters.

In the current set of studies, we examined the extent to which explicitly quantifying how much alcohol each person consumed—and whether these were equivalent—before an otherwise ambiguous sexual encounter influenced third-party perceptions of whether the sex that occurred was consensual, and whether different perceptions emerged for same-gender dyads compared to mixed-gender ones.

## Alcohol Consumption and Sexual Assault

The co-occurrence of alcohol consumption and sexual assault has been studied frequently, with alcohol being seen as adding to the risk of perpetration and victimization. In a 90-day diary study of men in college, Shorey and colleagues (2014) found that as the number of alcoholic drinks consumed increased, so too did the odds of physical and sexual aggression against a partner. This increase was not seen for other substances commonly used by young adults in social settings such as marijuana. In a review of studies comparing the characteristics of sexual assault perpetrators who had consumed alcohol and those who had not prior to their assaults, Abbey and colleagues (2001) found that perpetrators were generally hostile toward women, held traditional gender role beliefs, and occasionally, were victims of sexual abuse themselves. They concluded that perpetrators of sexual assault, regardless of whether the incident occurred following alcohol consumption, were more similar to each other than they were to men who had not committed sexual assaults. However, one difference that did emerge was that alcohol-fueled perpetrators reported greater alcohol consumption during sexual encounters and believed that a woman’s drinking signaled sexual interest (Brown et al., 2016; Zawacki et al., 2003). Thus, alcohol consumption does not necessarily directly lead to perpetration, despite the apparent link between alcohol and sexual assault.

Despite the perceived pathways between alcohol consumption and the *perpetration* of sexual assault (Abbey et al., 2022; Grubb & Turner, 2012; Melkonian & Ham, 2018; Steele & Josephs, 1990; Tuliao & McChargue, 2014), the society also continues to hold fraught ideas regarding intoxicated *victims* of sexual assault. Alcohol consumption and intoxication, as well as casual sex, are seen as less acceptable for women compared to men (Farvid & Braun, 2018; Norris, 1994), and women who drink are perceived as more promiscuous and sexually available than women who do not drink (Abbey et al., 1996; Ellis, 2019). Interestingly, the propensity to blame female victims of gendered violence who had consumed alcohol prior to the incident does not appear to extend to all forms of intoxication, such as prescription drug use (Sáez et al., 2020), suggesting that the associations between victim blaming and intoxication are partly informed by social mores surrounding specific intoxicants. The association between victim intoxication and greater perceived victim culpability is consistent with other work suggesting that women who violate traditional gender norms (e.g., drink in excess) are seen as more blameworthy for their attacks than women who conform to gender roles and do not drink (Grubb & Turner, 2012). In many instances, victim intoxication is not even a requirement; accepting even a single drink from a potential aggressor leads to greater attributions of blame placed on a female victim than those who reject the drink (Romero-Sánchez et al., 2018).

## Contextual Ambiguity as a Barrier to Acknowledging Assault

In theory, sexual assault is an unambiguous event: people either consent to have sex or they do not (Deming et al., 2013). In reality, people struggle to label these experiences, both as someone who may or may not have experienced an assault (Kahn et al., 2003; Peterson & Muehlenhard, 2011), and as third-party observers (Lamarche & James-Hawkins, 2022). Research from Lamarche and James-Hawkins (2022) found that individuals rely on contextual cues about the situation (i.e., who was intoxicated, how much could be remembered) to determine whether ambiguous sexual encounters were coercive or inappropriate. However, consistent with past research on unacknowledged sexual assault (Littleton & Axsom, 2003; Littleton et al., 2007, 2017), participants in Lamarche and James-Hawkins’ (2022) studies were also reluctant to go so far as to label events as sexual assaults even when they saw them as inappropriate, coercive, and even nonconsensual.

Qualitative data suggest that this reticence may stem in part from not knowing who is to blame when both sexual partners are intoxicated (James-Hawkins & Lamarche, forthcoming). For instance, although people agreed that an assault had likely occurred when only one sexual partner was intoxicated to the point of not remembering the evening, there was less agreement on what it meant when both sexual partners were *equally* drunk and therefore equally unable to consent. This is consistent with other qualitative research that has also identified that some people see intoxication parity as a way of handling the complexity of consent, and that it is when people are not equally drunk that the interaction becomes questionable (Hunt et al., 2021). Thus, although unequal intoxication levels may suggest a greater likelihood of harm (i.e., a drunk clubgoer forcing themselves on their sober dance partner; an intoxicated student being preyed on by an opportunistic perpetrator), equally high levels of intoxication may contribute to greater ambiguity and uncertainty of just who is the perpetrator and who is the victim.

## Gender, Sexual Orientations, and Consent

The gender of victims and perpetrators plays a recurring moderating role in the majority of research on sexual victimization. Moral typecasting leads people to assign men the roles of perpetrators and women the roles of victims (Reynolds et al., 2020). People are also more likely to attribute fewer masculine features, and more feminine features, to sexual assault victims regardless of gender (Mulder et al., 2020). Also, people find claims of sexual harassment more credible when the female victim is a more prototypical women (i.e., feminine appearance and interests) than when she is a non-prototypical woman (Goh et al., 2021). Gender differences also emerge in attributions of blame and acceptance of sexual assault; men are more likely to endorse rape myths and are more likely to place blame on victims compared to women, although women who endorse more traditional gender roles show parallel attitudes to men (Grubb & Turner, 2012). Although most work focuses on women as victims, research that includes men has shown that people blame male and female victims for different reasons. Male victims are blamed for not fighting back or resisting when they are scared, whereas women are blamed for not being cautious enough in their interactions with men (Howard, 1984a, 1984b; Hlavka, 2017).

Furthermore, research on sexual victimization has historically focused on heteronormative sexual interactions and contexts. However, sexual assault affects sexual minority communities as well (e.g., McKie et al., 2020). A cross-sectional survey of 21,000 American college-students found that gay and bisexual men reported similar incidence rates of sexual assaults as heterosexual women, while bisexual women experienced the highest rates of assault (Ford & Soto-Marquez, 2016). Gay men are believed to have the highest incidences of unreported intimate partner violence due to heterosexist pressures and homophobia (Finneran et al., 2012), and consistent with the feminization of sexual assault victims, gay men who are seen as effeminate are more likely to be degraded and victimized (Hunt et al., 2016; Salvati et al., 2016). The associations between alcohol use and sexual assault are mixed for lesbian women, with some studies suggesting that lesbian women are less likely to experience alcohol-associated sexual assault in adulthood compared to heterosexual women (Hughes et al., 2001), and others suggesting that they are more likely to experience alcohol-associated sexual assaults (Gilmore et al., 2014). Thus, intersectional research considering how gender and sexuality interact to influence perceptions of alcohol-associated victimization is needed for greater inclusivity and a more nuanced understanding of this social issue.

## Current Research

The current research aimed to better understand how contextual information alters perceptions of alcohol-fueled sexual interactions. Specifically, we examined whether the explicitly stated quantity of alcohol consumed by each partner, and whether these amounts matched, as well as the gender and sexual orientations of the people involved in the sexual encounters, influence how coercive and consensual an interaction is perceived to be, as well as whether it was likely to be considered a sexual assault, and how accountable the person describing the scenario was for the outcome. It is clear from the existing literature that most people recognize the added risk alcohol consumption contributes to sexual assaults. Even still, ambiguity exists. Specifically, the fact that alcohol use is clearly linked with perpetration is somehow undermined by the belief that victims share the blame if they too are intoxicated (e.g., Hunt et al., 2021; James-Hawkins & Lamarche, forthcoming). Furthermore, in much of the extant survey research on this topic, it is difficult to separate alcohol use by the victim/perpetrator because both parties are often drinking (e.g., Zawacki et al., 2003; Pegram et al., 2018; Woerner et al., 2018). The current research addresses these methodological limitations by explicitly quantifying alcohol consumption, as well as whether quantities were matched or unmatched between partners, across scenarios. Studies 1 and 2 investigated these patterns within mixed-gender dating contexts, and Studies 3 and 4 extended them to same-gender dating contexts (man–man and woman–woman, respectively). The study materials, aggregate data and syntax for re-analysis are available on the project’s repository on the Open Science Framework (OSF; https://osf.io/w5nrk/).

## Study 1

Study 1 used a series of vignettes to explore how different levels of alcohol consumption (i.e., one shot versus many shots) and congruency of alcohol consumption across sexual partners (i.e., same amount of alcohol consumed vs. different amounts of alcohol consumed) affected how people perceived sexual interactions between men and women, and the blame placed on the women across scenarios. Consistent with perceptions of alcohol use during sexual assaults and gender stereotypes, we hypothesized that people would rate scenarios more negatively when greater quantities of alcohol were consumed (i.e., 15 shots vs. 1 shot), and when alcohol consumption was mismatched.

### Method

#### Participants

Sixty-seven participants (78% women) were recruited for this study. Approximately half (48%) of participants were psychology undergraduate students who completed the study for research credit, and the other half were uncompensated volunteers recruited through social media platforms. Participants had to be over the age of 18 to participate (*M_age_* = 27.597, *SD* = 10.617) and the majority identified as White (81%; Asian, 10%; Black, 5%; Multiple Ethnicities, 4%), as well as identified as straight (82%; bisexual 13%; gay/lesbian, 5%), and monogamous in their preferred relationship style (98%; other style preferred 2%). Sensitivity analyses in G*Power for repeated measure ANOVAs, using a significance criterion of α = .050, and a power criterion of 80% suggest that a sample size of 67 participants should be able to detect an effect size of *f* = .140 (η^2^_partial_ *=* .020).

#### Procedure

Eligible participants (i.e., over 18; those who agreed to give their best answers on an integrity check pre-survey) first completed demographic measures (i.e., age, gender, ethnicity) and personality measures unrelated to the current hypotheses. Participants were then presented with instructions that they were going to be asked to think about and answer questions about a series of scenarios that occurred following a night out (see the study materials on the project’s OSF). Participants read four scenarios in total adapted from Lamarche and James-Hawkins (2022). Presentation of the scenarios was randomized across all participants. Scenarios were crossed in terms of how much alcohol was consumed (1 shot or 15 shots) and whether both partners consumed the same quantity (matched or unmatched). In each scenario, each person either consumed 1 shot or 15 shots. Scenario 1 was a matched alcohol scenario, with the man consuming one shot and the woman consuming one shot. Scenario 2 was also a matched scenario, with the man having 15 shots and the woman having 15 shots. Scenario 3 was a mismatched scenario, where the woman had consumed 15 shots and the man had one shot. Scenario 4 was another mismatched scenario, where the man had consumed 15 shots and the woman had consumed 1 shot. Each scenario ended with the couple having sex that night.

##### Sample Scenario (1 shot each)

Tori was at a mate’s party and was having so much fun. Dan, a guy from her English lecture started talking to her. The conversation was flowing and the couple each had 1 shot together at the bar. Tori and Dan ended up leaving the party together and had sex.

Immediately following each scenario, participants were presented with 11 questions about their view on the situation, which included one of the target outcomes assessing how coercive the man had been in the scenario, followed by more assessments of the interaction unrelated to the current article. Finally, participants were asked to assess how consensual the sex had been, whether it described a sexual assault, and to complete a measure assessing the accountability of the woman for the events that transpired.

#### Measures

##### Coercion

Participants rated the sexual encounter across five domains including the target question of whether or not the man in the scenario had been coercive (1 = *completely disagree*, 7 = *completely agree*; adapted from Lamarche & James-Hawkins, 2022).

##### Consent Given

A single-item measure was used to determine whether consent had been given in the scenario described (1 = *definitely did not give consent*, 7 = *definitely gave consent;* Lamarche & James-Hawkins, 2022).

##### Sexual Assault

A single-item measure was used to determine whether the scenario described a sexual assault (1 = *definitely did not describe a sexual assault*, 7 = *definitely described a sexual assault*; Lamarche & James-Hawkins, 2022).

##### Perceived Responsibility Over Outcome

Participants were asked to identify the degree to which they agreed/disagreed with five statements assessing the woman’s perceived responsibility for the outcome of that scenario (0 = *strongly disagree*, 10 = *strongly agree*). The statements stated that they believed the woman was responsible for what happened to her, that she deserved it, that she had been careless, and that she recovered well after the incident (α = .880 for all items across scenarios; adapted from van Prooijen & van den Bos, 2009).

### Results

**Figure 1.**
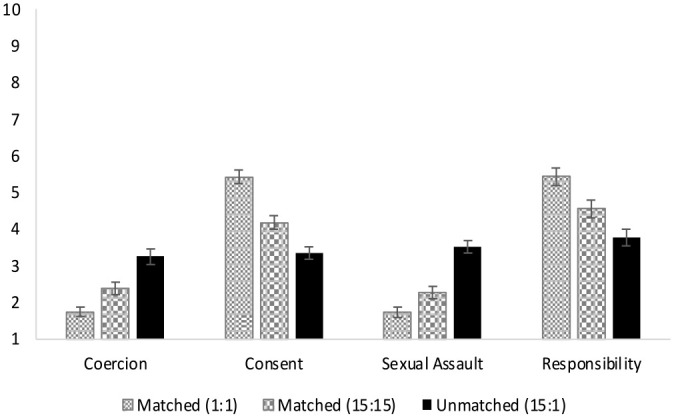
Mean ratings of coercion, consent, sexual assault, and victim blaming across scenarios in Study 1. *Note.* The *x*-axis captures the dependent variables and the *y*-axis the participant ratings for each DV. Each bar reflects the mean ratings for each DV per scenario described. Higher scores reflect greater endorsement that scenarios were coercive (1 = completely disagree, 7 = completely agree), consensual (1 = definitely did not give consent, 7 = definitely gave consent), and described a sexual assault (1 = definite did not describe a sexual assault, 7 = definitely described a sexual assault), and whether the woman was responsible (0 = strongly disagree, 10 = strongly agree).

Prior to analysis, the means of two of the unmatched scenarios were averaged for each outcome variable in order to make it possible to compare the unmatched scenarios (15:1 shots consumed) against the two matched scenarios (1:1 shots and 15:15 shots consumed).^1^ Repeated measures ANOVAs were used to predict (a) perceived coercion, (b) perceived consent, (c) describing a sexual assault, and (d) perceived responsibility for the outcome, from scenario (matched 1:1, matched 15:15, unmatched 15:1). Table 1 and Figure 1 present the mean ratings across scenarios. Ratings of perceived coercion, *F* (2,132) = 26.349, *p* *<* .001, η^2^_partial_ *=* .285, consent, *F* (2,132) = 60.987, *p* *<* .001, η^2^_partial_ *=* .480, sexual assault, *F* (2,132) = 40.025, *p* *<* .001, η^2^_partial_ *=* .378, and accountability *F* (2,132) = 26.055, *p* *<* .001, η^2^_partial_ *=* .283, all differed significantly across scenarios.

**Table 1.** Mean Ratings of Coercion, Consent, Sexual Assault, and Perceived Responsibility for Each Scenario in Study 1.

Scenario	Dependent Variables			
	Coercion *M* (*SD*)	Consent *M* (*SD*)	Sexual Assault *M* (*SD*)	Perceived Responsibility *M* (*SD*)
Matched, 1 shot each	1.746 (1.035)	5.418 (1.489)	1.731 (1.201)	5.436 (1.964)
Matched, 15 shots each	2.373 (1.369)	4.179 (1.497)	2.269 (1.399)	4.562 (1.979)
Unmatched, 15:1 shots	3.253 (1.737)	3.343 (1.404)	3.515 (1.474)	3.775 (1.878)

*Note.* Higher scores reflect greater endorsement that scenarios were coercive (1 = completely disagree, 7 = completely agree), consensual (1 = definitely did not give consent, 7 = definitely gave consent), described a sexual assault (1 = definite did not describe a sexual assault, 7 = definitely described a sexual assault), and that the woman was responsible for the outcome of that scenario (0 = strongly disagree, 10 = strongly agree).

Pairwise comparisons with Fisher’s least significant difference (LSD) were used to test for differences between scenarios. Table 2 presents the mean differences and 95% confidence intervals for differences for each comparison. All of the scenarios significantly differed from each other across all of the outcomes. As hypothesized, the scenario in which the couple were matched at a lower level of alcohol consumption (1:1 shots) was seen as the less coercive, more consensual and less likely an assault, but attributed *more* responsibility to the woman for the outcome of the interaction, compared to all other scenarios (*ps* ≤ .002), followed by the scenario in which the couple were matched at the higher level (15:15 shots) of alcohol consumption (*ps* ≤ .002). Notably, scenarios in which the couple were unmatched in alcohol consumption (15:1 shots) were seen as the most coercive, least consensual and most likely to describe an assault, and also ascribed the *least* responsibility to the woman compared to all other scenarios (*ps* ≤ .002).

**Table 2.** Pairwise Comparisons with Fisher’s Least Significant Difference for Study 1.

Dependent Variable	Scenario (*i*)		Scenario (*j*)	Mean Difference (*i–j*)	95% Confidence Interval	
					Lower	Upper
Coercion	Matched, 1 shot each	vs.	Matched, 15 shots each	−.627***	−0.950	−0.304
	Matched, 1 shot each	vs.	Unmatched, 15:1 shots	−1.507***	−1.933	−1.802
	Matched, 15 shots each	vs.	Unmatched, 15:1 shots	−.881***	−1.365	−0.396
Consent	Matched, 1 shot each	vs.	Matched, 15 shots each	1.239***	0.902	1.576
	Matched, 1 shot each	vs.	Unmatched, 15:1 shots	−2.075***	1.677	2.473
	Matched, 15 shots each	vs.	Unmatched, 15:1 shots	.836***	0.442	1.230
Sexual assault	Matched, 1 shot each	vs.	Matched, 15 shots each	−.537**	−0.867	−0.208
	Matched, 1 shot each	vs.	Unmatched, 15:1 shots	−1.784***	−2.217	−1.350
	Matched, 15 shots each	vs.	Unmatched, 15:1 shots	−1.246***	−1.698	−0.795
Perceived responsibility	Matched, 1 shot each	vs.	Matched, 15 shots each	.874***	0.472	1.276
	Matched, 1 shot each	vs.	Unmatched, 15:1 shots	1.661***	1.148	2.174
	Matched, 15 shots each	vs.	Unmatched, 15:1 shots	.787***	0.330	1.245

*Note.* ^†^*p* < .10. *p < .05. **p < .01. ***p < .001.

## Study 2

The aim of Study 2 was to replicate and extend the findings from Study 1. One possible explanation for the findings in Study 1 may be preexisting narratives regarding gender and intoxication. The mismatched scenarios may therefore have been evaluated more negatively because they reinforce the notion of a man as a perpetrator and a woman as a victim (i.e., a very drunk man who will ignore his relatively sober partner’s signals, or a relatively sober man taking advantage of a very drunk woman; Reynolds et al., 2020). In Study 2, the identical scenarios and questions were used as Study 1 except that the gender of the sexual partners was omitted, referring to each person in the scenarios and questions as Partner A (originally the women in Study 1) and Partner B (originally the men in Study 1). Following the scenarios, participants were further asked the extent to which they would assign one gender over another to each person in the scenario. This enabled us to test whether the pattern for mismatched alcohol is solely due to expectations regarding the gender of the partners.

### Methods

#### Participants

Sixty-eight participants (78% women) over the age of 18 were recruited to participate in this study (*M*_age_ = 31.132, *SD* = 14.782). Participants were undergraduate psychology students participating for research credits (36%) or volunteers recruited using social media platforms. The majority identified as White (80%; 7% Black; 3% Asian; 1% Latinx; 1% Middle-Eastern; 6% Multiple Ethnicities, or other), as well as straight (83%; 7% bisexual; 3% gay/lesbian; 3% not listed), and monogamous (91%; 4% another relationship style; 1% consensual nonmonogamy/polyamory). Sensitivity analyses in G*Power for repeated measure ANOVAs using a significance criterion of α = .050 and a power criterion of 80% suggest that a sample size of 68 participants should be able to detect an effect size of *f* = .140 (η^2^_partial_ *=* .020).

#### Procedure

Study 2 used the same methods and procedures as Study 1. However, in Study 2, the people in the scenarios were referred to as Person A (replacing the female-named person from Study 1) and Person B (replacing the male-named person; see OSF for scenarios). Thus, mirroring the scenarios from Study 1, in Scenario 1, both the focal partner of the scenario and their partner consumed 1 shot each; in Scenario 2, the focal partner and their partner consumed 15 shots each; in Scenario 3, the focal partner consumed 15 shots and their partner consumed 1 shot; and, in Scenario 4, the focal partner had 1 shot and their partner had 15. In addition to the measures of coercion, consent, sexual assault, and perceived responsibility from Study 1, participants were also asked to assign a gender to the focal partner and their partner using a continuous measure (0 = *male*, 10 = *female*).

### Results

**Figure 2.**
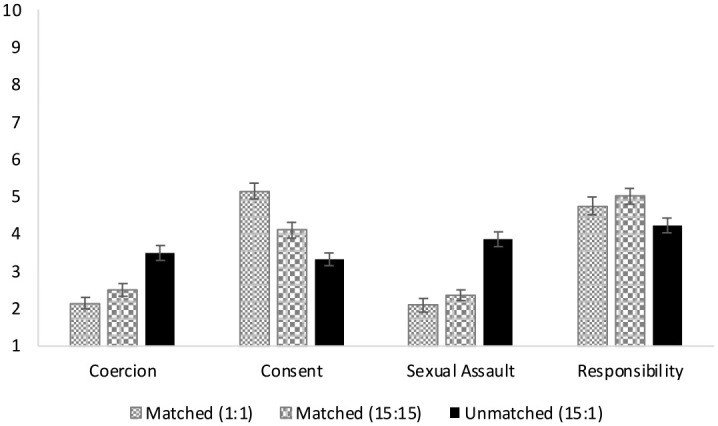
Mean ratings of coercion, consent, sexual assault, and perceived responsibility across scenarios in Study 2. *Note.* The *x*-axis captures the dependent variables and the *y*-axis the participant ratings for each DV. Each bar reflects the mean ratings for each DV per scenario described. Higher scores reflect greater endorsement that scenarios were coercive (1 = completely disagree, 7 = completely agree), consensual (1 = definitely did not give consent, 7 = definitely gave consent), and described a sexual assault (1 = definite did not describe a sexual assault, 7 = definitely described a sexual assault), and the perceived responsibility of the focal partner for the outcome of the scenario (0 = strongly disagree, 10 = strongly agree).

We used the same analytic approach for Study 2 as in Study 1. Table 3 and Figure 2 presents the mean ratings across scenarios. Consistent with Study 1, ratings of coercion, *F* (2, 134) = 24.814, *p* < .001, η^2^_partial_ *=* .270, consent, *F* (2, 134) = 36.809, *p* *<* .001, η^2^_partial_ *=* .355, sexual assault, *F* (2, 134) = 6.074, *p* *=* .003, η^2^_partial_ *=* .083, and perceived responsibility, *F* (2, 134) = 5.756, *p* = .004, η^2^_partial_ *=* .079, significantly differed across scenarios.

**Table 3.** Mean Ratings of Coercion, Consent, Sexual Assault, Perceived Responsibility, and Attributions of Gender for Each Scenario in Study 2.

Scenario	Dependent Variables					
	Coercion *M* (*SD*)	Consent *M* (*SD*)	Sexual Assault *M* (*SD*)	Perceived Responsibility *M* (*SD*)	Gender Partner A *M* (*SD*)	Gender Partner B *M*(*SD*)
Matched, 1 shot each	2.132 (1.280)	5.132 (1.761)	2.088 (1.552)	4.747 (1.918)	5.956 (2.975)	3.836 (2.858)
Matched, 15 shots each	2.500 (1.344)	4.103 (1.694)	2.353 (1.243)	5.006 (1.683)	5.500 (3.059)	4.328 (3.002)
Unmatched, 15:1 shots	3.463 (1.603)	3.316 (1.445)	2.860 (1.602)	4.234 (1.634)	6.574 (2.529)	3.231 (2.391)

*Note.* For gender of partners in scenarios, higher scores reflect greater confidence that the focal partner is a woman, lower scores a man (0 = male, 10 = female). For all other measures, higher scores reflect greater endorsement that scenarios were coercive (1 = completely disagree, 7 = completely agree), consensual (1 = definitely did not give consent, 7 = definitely gave consent), and described a sexual assault (1 = definite did not describe a sexual assault, 7 = definitely described a sexual assault), and the perceived responsibility of the focal partner for the outcome of the scenario (0 = strongly disagree, 10 = strongly agree).

However, the pairwise comparisons using LSD diverged slightly from Study 1. Table 4 presents the mean differences and 95% confidence intervals for differences for each comparison. Consistent with Study 1, the matched scenario in which both partners drank 1 shot was seen as less coercive and more consensual than all the other conditions (*ps* *<* .02). However, unlike in Study 1, ratings for the matched 1:1 scenario did not differ from the matched 15:15 scenario in when it came to evaluations of an assault and the responsibility of the focal partner (*ps* *>* .20). Consistent with Study 1, the unmatched scenarios were seen as the most coercive, least consensual, and most likely sexual assault than all the other scenarios (*ps* ≤ .022). Furthermore, consistent with Study 1, the focal partner in the unmatched scenarios was seen as more responsible for the outcome compared to the other scenarios (*ps* ≤ .017).

**Table 4.** Pairwise Comparisons with Fisher’s Least Significant Difference for Study 2.

Dependent Variable	Scenario (*i*)		Scenario (*j*)	Mean Difference (*i*−*j*)	95% Confidence Interval	
					Lower	Upper
Coercion	Matched, 1 shot each	vs.	Matched, 15 shots each	−.368*	−0.666	−0.069
	Matched, 1 shot each	vs.	Unmatched, 15:1 shots	−1.360***	−1.791	−0.930
	Matched, 15 shots each	vs.	Unmatched, 15:1 shots	−.993***	−1.443	−0.542
Consent	Matched, 1 shot each	vs.	Matched, 15 shots each	1.029***	0.579	1.480
	Matched, 1 shot each	vs.	Unmatched, 15:1 shots	1.816***	1.415	2.218
	Matched, 15 shots each	vs.	Unmatched, 15:1 shots	.787***	0.369	1.205
Sexual assault	Matched, 1 shot each	vs.	Matched, 15 shots each	−.265	−0.669	0.140
	Matched, 1 shot each	vs.	Unmatched, 15:1 shots	−.772**	−1.279	−0.265
	Matched, 15 shots each	vs.	Unmatched, 15:1 shots	−.507*	−0.938	−0.077
Perceived responsibility	Matched, 1 shot each	vs.	Matched, 15 shots each	−.259	−0.785	0.268
	Matched, 1 shot each	vs.	Unmatched, 15:1 shots	.514*	0.093	0.934
	Matched, 15 shots each	vs.	Unmatched, 15:1 shots	.772***	0.339	1.206

*Note.* ^†^*p* < .10. *p < .05. **p < .01. ***p < .001.

Finally, we tested whether people were more likely to attribute specific genders to the focal partners and their partners, collapsing across scenarios. People were significantly more likely to believe that the focal partners in the scenarios were women (*M* = 6.151, *SD* = 2.165) and that their partners were men (*M* = 3.676, *SD* = 1.948), *F* (1, 67) = 25.333, *p* < .001, η^2^_partial_ *=* .274.

## Studies 3 and 4

Studies 1 and 2 provided preliminary evidence that quantifiable alcohol consumption, particularly information which illustrates that consumption was unmatched, influences how people evaluate alcohol-fueled sexual interactions. However, the findings from Study 2 suggest that even when gender was omitted, people still applied gendered assumptions about the focal partners in a pattern consistent with a heterosexual hook-up. This suggests that people use a heterocentric lens when making decisions about sexual encounters even without explicit information supporting these assumptions. Given that gender informs perceptions of sexual interactions independent of other contextual information, and that the influence of heteronormative gender dynamics could not be ruled out by Study 2 as participants still made gendered assumptions about the people in the scenarios, it is important to extend these findings to same-sex interactions to see how people process differences in intoxication when gender imbalances in power cannot as obviously manifest. The aim of Studies 3 and 4 was to test whether the overall pattern of findings from Studies 1 and 2 could be replicated when the partners were presented as two men (Study 3) and as two women (Study 4). The methods for both studies were identical. We therefore present the methods and results together for comparison.

### Methods

#### Participants

Participants in both studies were recruited using Prolific Academic. Prolific is an online recruitment platform based in the UK, which allows researchers to post links to their studies and pay the participants directly via the platform without needing to exchange personalized or individuated information. Prolific requires researchers pay participants a minimum rate of £6.00 GBP/h. Participants in Studies 3 and 4 therefore received £2.00 for their participation in a 20-min study. Sensitivity analyses in G*Power for repeated measure ANOVAs using a significance criterion of α = .05 and a power criterion of 80% suggest that the samples in each study should be able to detect an effect size of *f* = .080 (η^2^_partial_ *=* .010).

In Study 3, we recruited 206 adults living in the UK (49% men, 50% women, and 1% other identity not listed) between the ages of 18 and 49 (*M_age_* = 29.880, *SD* = 7.944) who completed the survey to the end. The majority identified as White (74%; 16% Asian; 6% Black; 1% Latinx; 1% Middle-eastern; 1% Indigenous; 1% mixed ethnic group or other identity not listed). Most participants identified as straight (84%; 4% bisexual; 11% gay/lesbian; 1% not listed) and most identified as typically monogamous (97%; 2% consensual nonmonogamy/polyamory; 1% another relationship style).

In Study 4, we recruited 198 participants (50% women) between the ages of 18 and 49 (*M_age_* = 27.294, *SD* = 7.738) who completed the survey to the end. The majority identified as White (84%; 4% Asian; 2% Middle-Eastern; 2% Latinx; 1% Black; 7% mixed or multiple ethnicities or ethnicity not listed). Most participants identified as straight (80%; 13% bisexual; 5% gay/lesbian; 2% not listed) and most identified as typically monogamous (95%; 4% consensual nonmonogamy/polyamory; 1% another relationship style).

#### Procedures

Both studies used the same measures and procedures as Study 1, except this time depicted same-gender couplings in each study. In Study 3 both partners had stereotypically British male names, and in Study 4 both partners had stereotypically British female names.

### Results

**Figure 3.**
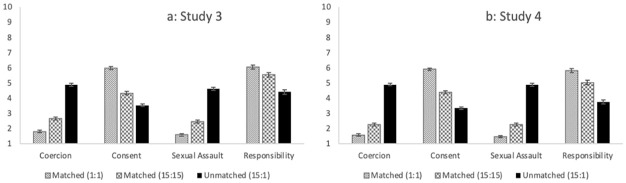
Mean ratings of coercion, consent, sexual assault, and perceived responsibility across in Studies 3 and 4. *Note.* The *x*-axis captures the dependent variables and the *y*-axis the participant ratings for each DV. Each bar reflects the mean ratings for each DV per scenario described. Higher scores reflect greater endorsement that scenarios were coercive (1 = completely disagree, 7 = completely agree), consensual (1 = definitely did not give consent, 7 = definitely gave consent), and described a sexual assault (1 = definite did not describe a sexual assault, 7 = definitely described a sexual assault), and the perceived responsibility of the focal partner for the outcome of the scenario (0 = strongly disagree, 10 = strongly agree).

The same analytic strategy was used for Studies 3 and 4 as the previous studies. Table 5 and Figure 3 contain the mean scores across scenarios for Studies 3 and 4.^2^

**Table 5.** Mean Ratings of Coercion, Consent, Sexual Assault, and Perceived Responsibility for Each Scenario in Studies 3 and 4.

Study	Scenario	Dependent Variables			
		Coercion *M* (*SD*)	Consent *M* (*SD*)	Sexual Assault *M* (*SD*)	Perceived Responsibility *M* (*SD*)
Study 3	Matched, 1 shot each	1.801 (1.187)	5.976 (1.338)	1.582 (1.152)	6.0437 (2.057)
	Matched, 15 shots each	2.646 (1.464)	4.330 (1.702)	2.447 (1.409)	5.536 (2.267)
	Unmatched, 15:1 shots	4.876 (1.390)	3.527 (1.103)	4.612 (1.540)	4.408 (2.151)
Study 4	Matched, 1 shot each	1.571 (1.114)	5.899 (1.286)	1.460 (0.876)	5.805 (1.891)
	Matched, 15 shots each	2.263 (1.341)	4.389 (1.566)	2.263 (1.279)	5.025 (2.066)
	Unmatched, 15:1 shots	4.894 (1.234)	3.341 (1.088)	4.900 (1.367)	3.743 (1.819)

*Note.* Higher scores reflect greater endorsement that scenarios were coercive (1 = completely disagree, 7 = completely agree), consensual (1 = definitely did not give consent, 7 = definitely gave consent), described a sexual assault (1 = definite did not describe a sexual assault, 7 = definitely described a sexual assault), and the perceived responsibility of the focal partner for the outcome of the scenario (0 = strongly disagree, 10 = strongly agree).

#### Study 3

Ratings of perceived coercion, *F* (2,410) = 336.215, *p* < .001, η^2^_partial_ *=* .621, consent, *F* (2,410) = 280.845, *p* < .001, η^2^_partial_ *=* .578, sexual assault, *F* (2,410) = 372.248, *p* < .001, η^2^_partial_ *=* .645, and, perceived responsibility, *F* (2,410) = 92.119, *p* *<* .001, η^2^_partial_ *=* .310, all differed significantly across scenarios (Figure 3). The pairwise comparisons using LSD were consistent with Study 1 across all outcomes (see Table 6). Specifically, the matched scenario in which both men consumed 1 shot each was seen as the less coercive, more consensual, and less likely to be an assault, but also attributed the most responsibility to the focal partner (*ps* < .001) compared to the other scenarios, followed by the scenario in which both men had 15 shots (*ps* *<* .001). Similarly, the unmatched scenarios in which the one man drank 15 shots and one drank 1 shot were seen as the most coercive, least consensual, most likely to be an assault, but also least responsible compared to the other scenarios (*ps* *<* .001).

**Table 6.** Pairwise Comparisons with Fisher’s Least Significant Difference for Studies 3 and 4.

Study	Dependent Variable	Scenario (i)		Scenario (j)	Mean Difference (i−j)	95% Confidence Interval	
						Lower	Upper
Study 3	Coercion	Matched, 1 shot each	vs.	Matched, 15 shots each	−.845***	−1.062	−0.627
		Matched, 1 shot each	vs.	Unmatched, 15:1 shots	−3.075***	−3.330	−2.820
		Matched, 15 shots each	vs.	Unmatched, 15:1 shots	−2.231***	−2.481	−1.980
	Consent	Matched, 1 shot each	vs.	Matched, 15 shots each	1.646***	1.423	1.868
		Matched, 1 shot each	vs.	Unmatched, 15:1 shots	2.449***	2.266	2.632
		Matched, 15 shots each	vs.	Unmatched, 15:1 shots	.803***	0.588	1.019
	Sexual assault	Matched, 1 shot each	vs.	Matched, 15 shots each	−.864***	−1.071	−0.657
		Matched, 1 shot each	vs.	Unmatched, 15:1 shots	−3.029***	−3.275	−2.783
		Matched, 15 shots each	vs.	Unmatched, 15:1 shots	−2.165***	−2.387	−1.943
	Perceived responsibility	Matched, 1 shot each	vs.	Matched, 15 shots each	.508***	0.267	0.748
		Matched, 1 shot each	vs.	Unmatched, 15:1 shots	1.636***	1.382	1.890
		Matched, 15 shots each	vs.	Unmatched, 15:1 shots	1.128***	0.894	1.363
Study 4	Coercion	Matched, 1 shot each	vs.	Matched, 15 shots each	−.692***	−0.852	−0.532
		Matched, 1 shot each	vs.	Unmatched, 15:1 shots	−3.323***	−3.365	−3.081
		Matched, 15 shots each	vs.	Unmatched, 15:1 shots	−2.631***	−2.886	−2.377
	Consent	Matched, 1 shot each	vs.	Matched, 15 shots each	1.510***	1.277	1.743
		Matched, 1 shot each	vs.	Unmatched, 15:1 shots	2.558***	2.351	2.766
		Matched, 15 shots each	vs.	Unmatched, 15:1 shots	1.048***	0.807	1.289
	Sexual assault	Matched, 1 shot each	vs.	Matched, 15 shots each	−.803	−0.986	−0.620
		Matched, 1 shot each	vs.	Unmatched, 15:1 shots	−3.437	−3.665	−3.208
		Matched, 15 shots each	vs.	Unmatched, 15:1 shots	−2.634	−2.850	−2.417
	Perceived responsibility	Matched, 1 shot each	vs.	Matched, 15 shots each	.780	0.525	1.035
		Matched, 1 shot each	vs.	Unmatched, 15:1 shots	2.062	1.816	2.308
		Matched, 15 shots each	vs.	Unmatched, 15:1 shots	1.282	1.059	1.506

*Note.* ^†^*p* < .10. *p < .05. **p < .01. ***p < .001.

#### Study 4

Ratings of perceived coercion, *F* (2, 394) = 482.380, *p* < .001, η^2^_partial_ *=* .710, consent, *F* (2,394) = 248.281, *p* *<* .001, η^2^_partial_ *=* .558, sexual assault, *F* (2,394) = 568.151, *p* < .001, η^2^_partial_ *=* .743, and perceived responsibility, *F* (2,394) = 144.228, *p* < .001, η^2^_partial_ *=* .423, significantly differed across scenarios (Figure 3). The pairwise comparisons were consistent with Study 1 across all outcomes (see Table 6). Specifically, the matched scenario in which both women consumed 1 shot each was seen as the less coercive, more consensual, and less likely to be an assault, but also attributed the most responsibility to the focal partner (*ps* < .001) compared to the other scenarios, followed by the scenario in which both women had 15 shots (*ps* *<* .001). Similarly, the unmatched scenarios in which one woman drank 15 shots and one drank 1 shot were seen as the most coercive, least consensual, most likely to be an assault, but ascribed the least responsibility compared to the other scenarios (*ps* *<* .001).

## Discussion

The current research examined how quantifiable differences in alcohol consumption, and specifically the matching or mismatching of alcohol consumption, influenced perceptions of sexual interactions. Across four studies, consistent effects emerged regarding how quantifiable differences and similarities in alcohol consumption influence perceptions of alcohol-fueled sexual interactions. In all four studies, participants consistently evaluated the scenarios with mismatched alcohol consumption as more problematic (less consensual, more coercive, and more likely an assault) than the scenarios in which alcohol consumption was matched. This was true even relative to the condition in which both partners in the scenario consumed the same large quantity of alcohol (15 shots each), which differed from the other scenarios (matched 1 shot each, unmatched shots 15:1). These findings are consistent with past work which illustrates how alcohol consumption is an important contextual factor that influences perceptions of sexual receptivity (Farris et al., 2010; Romero-Sánchez et al., 2012). These findings are also consistent with qualitative data, which suggests that people struggle to label sexual experiences as nonconsensual when *both* sexual partners are intoxicated (Hunt et al., 2021; James-Hawkins & Lamarche, forthcoming). The current findings also illustrate the importance explicitly quantifying alcohol consumption can have at influencing perceptions of sexual encounters. Historically, most research treats intoxication as an abstract concept, typically mentioning that one or both people were “drunk” but without quantifying what that may mean, despite intoxication being something people experience incrementally rather than as a binary (Zajdow & MacLean, 2014). As suggested by our findings, people may perceive consent, coercion, sexual assault, and victim responsibility differently depending on how drunk each person is, which makes understanding consent even more challenging in alcohol-fueled contexts.

Another interesting pattern in our data emerged regarding the perceived responsibility of the focal partner in the scenario for the outcome of the interaction. Notably, although the scenario in which both partners drank only a small amount of alcohol was seen more positively, the focal partner of those scenarios was also seen as the most responsible for the outcome of that interaction compared to the focal partners in the other scenarios. By contrast, when there was a mismatch between how much the focal partner and their partner had consumed, the focal partners described in the scenarios were seen as the least responsible for the outcome relative to the matched alcohol consumption conditions. Thus, unlike in previous research where (feminine-presenting) people who had experienced sexual assault or harassment were blamed for getting drunk (Grubb & Turner, 2012; Romero-Sánchez et al., 2018), people in our studies seemed to acknowledge that mismatched alcohol consumption created riskier sexual interactions in which someone may have less autonomy over the outcome regardless of gender. Again, given that people ascribe potentially different motivations and expectations across the continuum of drunkenness, future research focused on victim blaming and the perceptions of perpetrators should consider whether or not they are explicitly labeling the quantity of alcohol consumed by each person and how that may influence perceptions.

Finally, it is notable to mention that these findings were consistent across studies, including both explicit (Study 1) and perceived (Study 2) mixed-gender hookups, and same-gender hookups between men (Study 3) and women (Study 4). Thus, despite different narratives centering men as “perpetrators” and women as “victims” (Reynolds et al., 2020), and different prevalence rates of sexual violence across demographics (Ford & Soto-Marquez, 2016), the people in our studies which included both men and women, and people with different sexual orientations, evaluated these scenarios consistently regardless of the (perceived) gender or sexuality of the people described.

### Limitations and Future Directions

Despite the strength of the current project, there are also limitations that need addressing in future research. First, the current project illustrates that people reliably perceive interactions in contexts in which one partner has consumed alcohol than the other as a better indicator of sexual misconduct than simply whether a lot of alcohol had been consumed, particularly in sexually ambiguous scenarios (i.e., instances in which sex has occurred but information about the emotional impact and desire associated is neutral; Lamarche & James-Hawkins, 2022). However, the current studies did not explore the psychological mechanisms that led to these judgments. For instance, it is possible that people assume a mutuality of intent or coordination in situations in which both people maintain the same level of intoxication, compared to situations in which one person drinks a lot more than the other. This suggests that people may erroneously project shared expectations onto people in situations in which their overt behaviors are congruent, but their internal psychological states (e.g., actual willingness to have sex) may nonetheless differ. Notably, it may perpetuate rape myths that if both people got drunk then they both wanted any sex that followed. Alternatively, it may also be the case that mismatched intoxication implies either calculated ill-intent (e.g., a relatively sober partner taking advantage of the drunk partner), or an inability to inhibit oneself due to intoxication (e.g., a drunk person missing overt body-language signaling disinterest in their relatively sober partner) which both create opportunities in which sexual misconduct can occur, but culpability is diminished. Thus, future research should aim to investigate the psychological mechanisms that underly the perceptions of matched/unmatched intoxication, particularly in sexually ambiguous encounters.

Second, the current project included diverse samples of participants from different ethnicities, sexual orientations, genders, and ages. Despite the ethnic diversity of this sample, the majority of the participants across the four studies were White adults living in the UK. Much of the previous work on alcohol-fueled sexual encounters has used American college samples. Thus, a UK sample helps address the generalizability of prior work by exploring these issues in a new, yet similar, context. The UK and the US have similar drinking cultural typographies (Savic et al., 2016), and sociocultural problems associated with alcohol beyond sexual assault, such as the socioeconomic cost of workplace hangovers and intoxication (Bhattacharya, 2019; Hickey, 2014). The UK drinking context also differs from the US, with both male and female undergraduate students in the UK engaging in more binge-drinking than their U.S. counterparts (Gill, 2002), the UK’s increasing drinking rate and peer pressure in young people have been blamed for this (Morris et al., 2020). Thus, future research should replicate these findings in other cultural contexts where the social context and expectations around alcohol consumption may differ.

Another limitation is that the majority of participants across our studies were between the ages of 18 and 30. People in this age range are more likely to expose themselves to information online, specifically through social media, with an estimated 67% of 18- to 29-year-olds using Instagram, 86% using Facebook, and 38% using Twitter (Khoros, 2021). Social media has provided a platform for individuals to share experiences of sexual misconduct and assault. An example is the 2017 “Me Too” movement that had a huge social media presence. “Me Too” exposed and educated social media users on the realities of sexual assault (Caputi et al., 2019). It is possible that if this study was open to participants of wider age ranges, the findings may be different. For example, individuals who have had less exposure to information regarding sexual assault may not show gender bias in who is a perpetrator and who is a victim. Similarly, older populations may hold more rigid rape scripts and rape myths compared to a younger generation who have grown up with more public discussion around consent and sexual assault. For example, more traditional views that lead people to believe that an intoxicated victim shares the blame with the perpetrator for what happens to them should lead to higher endorsements of victim blaming in the scenario in which the focal partner has more drinks than their partner. However, in our samples we saw the opposite pattern, suggesting a recognition that perpetrators must recognize when their partners are incapable of granting consent (e.g., when they are much more intoxicated).

### Diversity

Methodologically, our studies support diversity in social science research by presenting sexual encounters for both mixed-gender and same-gender sexual pairings. This enriches our understanding of how contextual information regarding (perceived) gender/sexual orientation may influence how people interpret ambiguous alcohol-fueled sexual encounters. Our samples included men and women living in the UK. As described above, this is a relatively unique sample as most research on alcohol-fueled sexual encounters focuses on American college-students. Studies 1 and 2 relied on an opportunity sample of UK adults, while Studies 3 and 4 relied on convenience samples of UK adults recruited through an online platform. Although we did not recruit nationally representative samples, our samples were consistent with the 2011 UK Census that reports approximately 80% of people in England and Wales identify as White, 7% as Asian, and 3% as Black. However, because the non-White samples across studies were relatively small it was not possible to test for cultural and/or ethnic differences. Similarly, our studies somewhat overrepresented participants from sexual minorities relative to nation-level data which suggests that, as of 2020, 92% of people 16 to 50 years old^3^ in the UK identify as heterosexual/straight, compared to only 2% as gay/lesbian, 2% as bisexual and 4% as other or refuse to say (Sharfman & Cobb, 2022). However, there was still limited power to test for differences as a function of sexual orientation. Our sample included people who were single and romantically attached, as well as those engaged in different relationship styles (e.g., monogamy, consensual nonmonogamy). As noted above, we had pragmatic reasons for restricting participation to adults between the ages of 18 and 50. However, these restrictions mean that some caution should be used when generalizing our findings across people of different ages.

## Conclusion

Sex never occurs in a contextual vacuum. The findings from the current research broaden our understanding of how people make sense of alcohol-fueled sexual encounters and their potential associations with sexual assault. Importantly, contextual information interacts with shape perceptions: people not only rely how much alcohol someone consumed prior to a sexual encounter, but more importantly whether partners were equally drunk.

## Supplemental Material

sj-docx-1-jiv-10.1177_08862605231182378 – Supplemental material for Just One Shot?: The Contextual Effects of Matched and Unmatched Intoxication on Perceptions of Consent in Ambiguous Alcohol-fueled Sexual Encounters Click here for additional data file. Supplemental material, sj-docx-1-jiv-10.1177_08862605231182378 for Just One Shot?: The Contextual Effects of Matched and Unmatched Intoxication on Perceptions of Consent in Ambiguous Alcohol-fueled Sexual Encounters by Ellen Laughlin, Molly Pettitt, Veronica M. Lamarche and Laurie James-Hawkins in Journal of Interpersonal Violence

## Notes

1. For transparency, the analyses comparing all four scenarios without collapsing across the unmatched scenarios for all studies are available in our online supplemental materials.

2. With the relatively larger samples in Studies 3 and 4 we were able to test, post hoc, whether gender and sexual orientation moderated our findings. In Study 3, sexual orientation moderated the effects for coercion, assault, and victim blaming (*ps* ≤ .020). Follow-up *t*-tests looking at scenario ratings by sexual orientation found sexual minority participants rated the unmatched scenario as more coercive and more likely an assault, and rated the focal partner in the unmatched scenario as less responsible compared to straight participants (*ps* < .020). However, these results should be interpreted with caution as this moderation was not predicted a priori and there were far fewer sexual minority participants in this sample compared to straight participants (33:177). This pattern did not replicate in Study 4 (*ps* *>* .098). Gender did not moderate our findings in either study (*ps* *>* .08).

3. The latest available data on sexual orientation by age in the UK reports data in subsets of 16 to 24, 25 to 34, and 34 to 49 years old, whereas our study was limited restricted to anyone under 18. However, given that sexual minority identities were most prevalent among 16- to 24-year-olds in 2020, our sample still likely over-represents sexual minority groups relative to baseline population rates.
