# Supplementary material for: Just One Shot? The Contextual Effects of Matched and Unmatched Intoxication on Perceptions of Consent in Ambiguous Alcohol-fueled Sexual Encounters
Source: J Interpers Violence. 2023 Jul 10;38(21-22):11445–74. doi: 10.1177/08862605231182378 (PMC10515474; doi:10.1177/08862605231182378)
Supplement: sj-docx-1-jiv-10.1177_08862605231182378 – Supplemental material for Just One Shot?: The Contextual Effects of Matched and Unmatched Intoxication on Perceptions of Consent in Ambiguous Alcohol-fueled Sexual Encounters [file sj-docx-1-jiv-10.1177_08862605231182378.docx]

**Table of Contents**

Results Study 1 Without Collapsing Across Condition 2

Table S1 Mean ratings across all 4 conditions Study 1 3

Figure S1 Mean ratings across all 4 conditions Study 1 4

Results Study 2 Without Collapsing Across Condition 5

Table S2 Mean ratings across all 4 conditions Study 2 6

Figure S2 Mean ratings across all 4 conditions Study 2 7

Results Study 3 Without Collapsing Across Condition 8

Table S3 Mean ratings across all 4 conditions Studies 3 & 4 9

Figure S3 Mean ratings across all 4 conditions Study 3 10

Results Study 4 Without Collapsing Across Condition 11

Figure S4 Mean ratings across all 4 conditions Study 4 12

**Results Study 1 Without Collapsing Across Condition**

Repeated measures ANOVAs were used to predict a) perceived coercion, b) perceived consent, c) describing a sexual assault, and d) victim blaming from scenario (matched 1:1, matched 15:15, unmatched 15:1). Ratings of perceived coercion, *F* (3,198) = 22.39, *p<*.001, *η^2^_partial_=*.25, consent, *F* (3,198) = 67.50, *p<*.001, *η^2^_partial_=*.51, sexual assault, *F* (3,198) = 52.01, *p<*.001, *η^2^_partial_=*.44, and victim blaming *F* (3,198) = 27.46, *p<*.001, *η^2^_partial_=*.29, all differed significantly across scenarios (Table S1 & Figure S1). Pairwise comparisons were used to test for differences between each scenario. As hypothesized, the scenario in which the woman and the man consumed 1 shot each was seen as the less coercive, more consensual and less likely an assault, but attributed *more* blame to the woman for the outcome of the interaction, compared to all other scenarios (*ps*<.02). Additionally, the scenarios where the woman drank more than the man was seen as the most coercive, least consensual and most likely an assault, and also ascribed the *least* blame to the female victim compared to all other scenarios (*ps<*.001). It is notable, however, that the scenario in which both the man and the woman consumed 15 shots, and the scenario in which the woman drank less than the man did not differ across any of the outcomes (*ps*>.11).

| **Table S1.**  *Mean ratings of coercion, consent, sexual assault and victim blaming for each scenario.* | | | | |
| --- | --- | --- | --- | --- |
|  | **Dependent Variables** | | | |
| **Scenario** | Coercion  M (SD) | Consent  M (SD) | Sexual Assault  M (SD) | Victim Blaming  M (SD) |
| 1: Woman 1 shot, Man 1 shot | 1.75 (1.04) | 5.42 (1.49) | 1.73 (1.20) | 5.44 (1.96) |
| 2: Woman 15 shots, Man 15 shots | 2.37 (1.37) | 4.18 (1.50) | 2.27 (1.40) | 4.56 (1.98) |
| 3: Woman 15 shots, Man 1 shot | 3.67 (2.06) | 2.16 (1.39) | 4.66 (1.94) | 2.88 (2.19) |
| 4: Woman 1 shot, Man 15 shots | 2.84 (1.94) | 4.52 (2.13) | 2.37 (1.83) | 4.67 (2.47) |
| ***Note.*** *Higher scores reflect greater endorsement that scenarios were coercive (1=completely disagree, 7=completely agree), consensual (1=definitely did not give consent, 7=definitely gave consent), and described a sexual assault (1=definite did not describe a sexual assault, 7=definitely described a sexual assault).* | | | | |

**Figure S1.**

*Mean ratings of coercion, consent, sexual assault and victim blaming across scenarios in Study 1.*


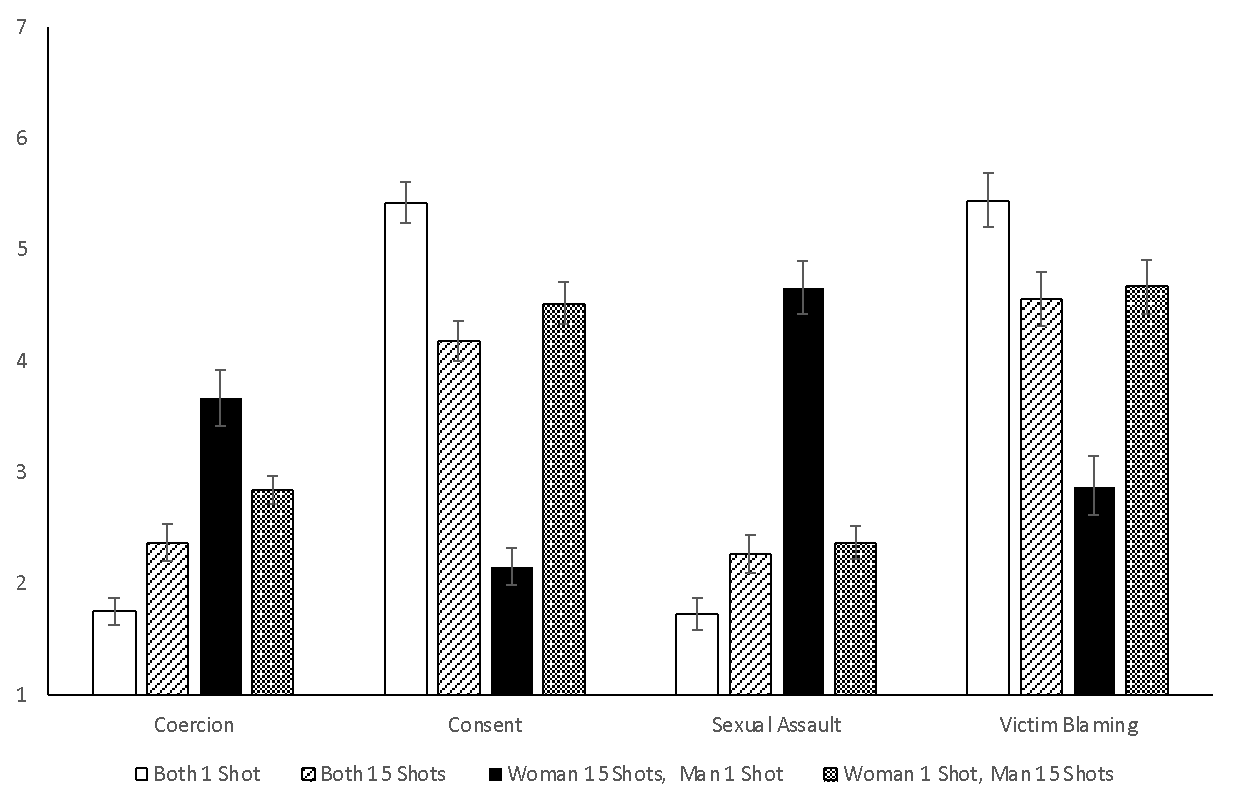


***Note.*** *The x-axis captures the dependent variables and the y-axis the participant ratings for each DV. Each bar reflects the mean ratings for each DV per scenario described.* *Higher scores reflect greater endorsement that scenarios were coercive (1=completely disagree, 7=completely agree), consensual (1=definitely did not give consent, 7=definitely gave consent), and described a sexual assault (1=definite did not describe a sexual assault, 7=definitely described a sexual assault), and whether the target was to blame for the outcome of the scenario (0=strongly disagree, 10=strongly agree).*

**Results Study 2 Without Collapsing Across Condition**

Repeated measures ANOVAs were again used to test for differences between scenarios in Study 2. Consistent with Study 1, ratings of coercion, *F*(3, 201) = 21.63, *p* < .001, *η^2^_partial_=*.24, consent, *F*(3, 201) = 37.55, *p <* .001, *η^2^_partial_=*.36, sexual assault*, F*(2, 201) = 6.05, *p <* .001, *η^2^_partial_=*.08, and victim blaming, *F*(3, 201) = 12.20, *p* < .001, *η^2^_partial_=*.15, significantly differed across scenarios (Table S2 and Figure S2). However pairwise comparisons diverged slightly from Study 1. The scenario in which both partners drank 1 shot was seen as less coercive and more consensual than all of the other conditions (*ps<*.02), but did not differ from the scenario in which both partners had 15 shots when it came to likelihood that the scenario depicted an assault and the blameworthiness of the target (*ps>*.20). The scenario in which the target had consumed 15 shots and their partner had consumed 1 (equivalent scenario to where the woman had 15 and the man had 1 in Study 1) was also seen as the most coercive, least consensual and most likely sexual assault than all the other scenarios (*ps<*.045), consistent with Study 1, but was seen as more blameworthy from the scenario in which the target consumed 1 shot and their partner 15 (equivalent scenario to where the woman had 1 and the man had 15 in Study 1*; p<*.001). Additionally, the scenarios in which both people had 15 shots and the one where the target had 1 shot compared to their partner who had 15 were no longer equivalent across outcomes, unlike in Study 1. Notably the scenario where both had 15 shots was seen as less coercive (*p<*.001), and more blameworthy (*p<*.001), but not less consensual or more likely an assault (*ps>*.22).

Finally, we tested whether people were more likely to attribute specific genders to the targets and their partners, collapsing across scenarios. People were significantly more likely to believe that the targets in the scenarios were women (*M*=6.15, *SD*=2.16) and that their partners were men (*M*=3.68, *SD*=1.94), *F*(1, 67) = 25.33, *p* < .001, *η^2^_partial_=*.27.

| **Table S2.**  *Mean ratings of coercion, consent, sexual assault, victim blaming and attributions of gender for each scenario.* | | | | | | |
| --- | --- | --- | --- | --- | --- | --- |
|  | **Dependent Variables** | | | | | |
| **Scenario** | Coercion  M (*SD*) | Consent  M (*SD*) | Sexual Assault  M (*SD*) | Victim Blaming  M (*SD*) | Gender Partner A M(*SD*) | Gender Partner B M(*SD*) |
| 1: Target 1 shot, Partner 1 shot | 2.13 (*1.28*) | 5.13 (*1.76*) | 2.09 (*1.55*) | 4.75 (.23) | 5.96 (*2.98*) | 3.84 (*2.86*) |
| 2: Target 15 shots, Partner 15 shots | 2.50 (*1.34*) | 4.10 (*1.69*) | 2.35 (*1.24*) | 5.01 (*.20*) | 5.50 (*3.06*) | 4.33 (*3.00*) |
| 3: Target 15 shots, Partner 1 shot | 3.82 (*1.86*) | 2.54 (*1.55*) | 3.09 (*2.04*) | 4.80 (.21) | 7.13 (*2.75*) | 2.64 (*2.57*) |
| 4: Target 1 shot, Partner 15 shots | 3.16 (*1.80*) | 4.09 (*2.06*) | 2.63 (*1.64*) | 3.67 (.24) | 6.01 (*3.26*) | 3.82 (*3.10*) |

***Note.*** *For gender of targets in scenarios, higher scores reflect greater confidence that the target is a woman, lower scores a man (0=male, 10=female). For all other measures, higher scores reflect greater endorsement that scenarios were coercive (1=completely disagree, 7=completely agree), consensual (1=definitely did not give consent, 7=definitely gave consent), and described a sexual assault (1=definite did not describe a sexual assault, 7=definitely described a sexual assault), and whether the target was to blame for the outcome of the scenario (0=strongly disagree, 10=strongly agree).*

**Figure S2.**

*Mean ratings of coercion, consent, sexual assault and victim blaming across scenarios in Study 2.*


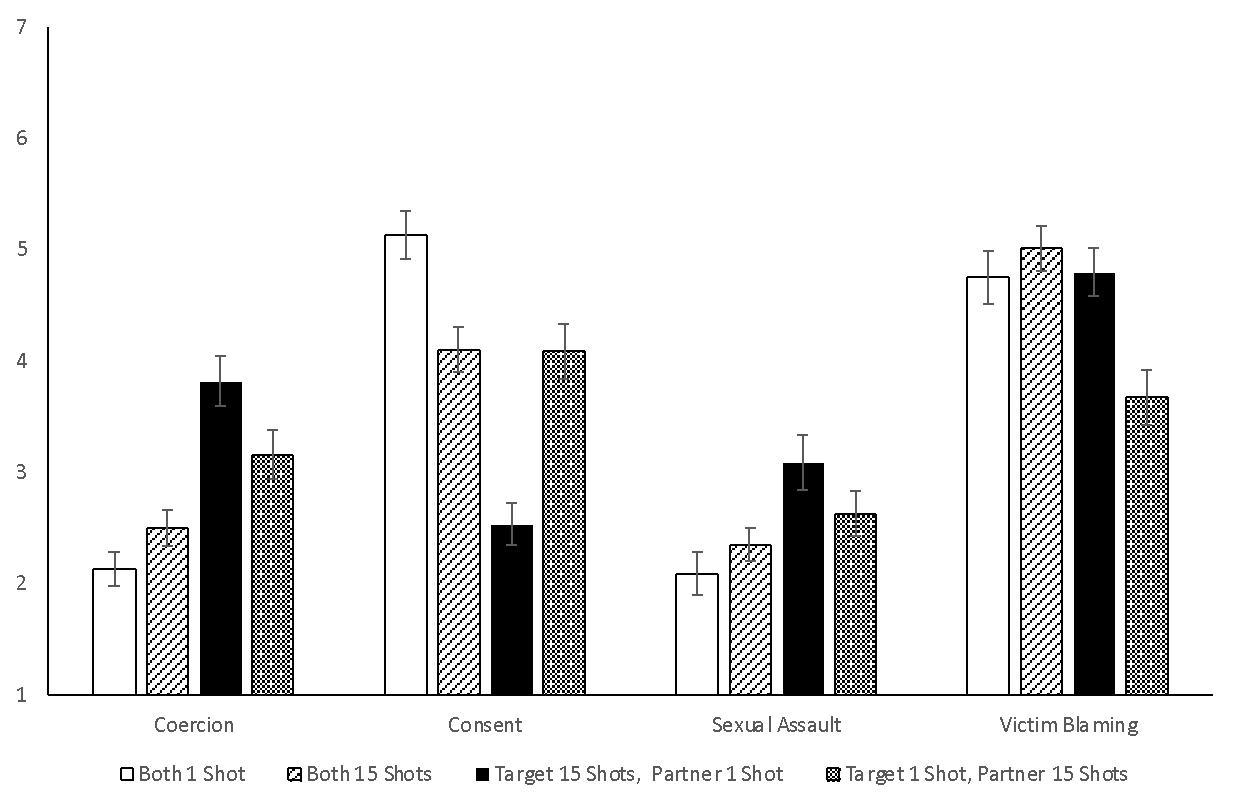


***Note.*** *The x-axis captures the dependent variables and the y-axis the participant ratings for each DV. Each bar reflects the mean ratings for each DV per scenario described.* *Higher scores reflect greater endorsement that scenarios were coercive (1=completely disagree, 7=completely agree), consensual (1=definitely did not give consent, 7=definitely gave consent), and described a sexual assault (1=definite did not describe a sexual assault, 7=definitely described a sexual assault), and whether the target was to blame for the outcome of the scenario (0=strongly disagree, 10=strongly agree).*

**Results Study 3 Without Collapsing Across Condition**

Ratings of perceived coercion, *F*(3,615) = 296.73, *p*<.001, *η^2^_partial_=*.59, consent, *F*(3,615) = 215.90, *p*<.001, *η^2^_partial_=*.52, sexual assault, *F*(3,615) = 338.52, *p*<.001, *η^2^_partial_=*.62, and, victim blaming *F*(3,615) = 125.32, *p<*.001, *η^2^_partial_=*.38, all differed significantly across scenarios (Table S3, Figure S3). The scenario in which both men consumed 1 shot each was seen as the less coercive, more consensual, less likely to be an assault, and most blameworthy (*ps*<.001) than all of the other scenarios, followed by the scenario in which both men consumed 15 shots each (*ps<*.001), and the scenario in which the target consumed 1 shot and his partner consumed 15, with the exception of victim blaming in which there was no difference between the scenario in which both consumed 15 shots and the scenario in which the target consumed 1 shot and his partner 15 when it came to perceptions of blame (*p=*.27). Furthermore, the scenario in which the target consumed 15 shots and his partner consumed 1 was seen as the most coercive, lease consensual, most likely to be an assault, and least blameworthy compared to the other scenarios (*ps<*.001).

| **Table S3.**  *Mean ratings of coercion, consent, sexual assault and victim blame for each scenario.* | | | | | |
| --- | --- | --- | --- | --- | --- |
|  | | **Dependent Variables** | | | |
| **Study** | **Scenario** | Coercion  M (*SD*) | Consent  M (*SD*) | Sexual Assault  M (*SD*) | Victim Blaming  M (*SD*) |
| Study 3:  Both Men | 1: Partner A 1 shot, Partner B 1 shot | 1.80 (*1.19*) | 5.98 (*1.39*) | 1.58 (*1.15*) | 6.04 (*2.06*) |
|  | 2: Partner A 15 shots, Partner B 15 shots | 2.65 (*1.46*) | 4.33 (*1.70*) | 2.45 (*1.41*) | 5.54 (*2.27*) |
|  | 3: Partner A 15 shots, Partner B 1 shot | 5.49 (*1.52)* | 1.85 (*1.20*) | 5.40 (*1.56*) | 3.35 (*2.33*) |
|  | 4: Partner A 1 shot, Partner B 15 shots | 4.27 (*1.79*) | 5.20 (*1.76*) | 3.82 (*1.99*) | 5.37 (*2.51*) |
| Study 4: Both Women | 1: Partner A 1 shot, Partner B 1 shot | 1.57 (*1.11*) | 5.90 (*1.29*) | 1.46 (.*88)* | 5.81 (*1.89*) |
|  | 2: Partner A 15 shots, Partner B 15 shots | 2.26 (*1.34*) | 4.39 (*1.57*) | 2.26 (*1.28*) | 5.03 (*2.07*) |
|  | 3: Partner A 15 shots, Partner B 1 shot | 5.49 (*1.38*) | 1.77 (*1.04*) | 5.52 (*1.47*) | 2.66 (*2.03*) |
|  | 4: Partner A 1 shot, Partner B 15 shots | 4.30 (*1.65*) | 4.91 (*1.87*) | 4.27 (*1.65*) | 4.82 (*2.29*) |
| ***Note.*** *Higher scores reflect greater endorsement that scenarios were coercive (1=completely disagree, 7=completely agree), consensual (1=definitely did not give consent, 7=definitely gave consent), and described a sexual assault (1=definite did not describe a sexual assault, 7=definitely described a sexual assault).* | | | | | |

**Figure S3.**

*Mean ratings of coercion, consent, sexual assault and victim blaming across scenarios with gay men in Study 3.*

***Note.*** *The x-axis captures the dependent variables and the y-axis the participant ratings for each DV. Each bar reflects the mean ratings for each DV per scenario described.* *Higher scores reflect greater endorsement that scenarios were coercive (1=completely disagree, 7=completely agree), consensual (1=definitely did not give consent, 7=definitely gave consent), and described a sexual assault (1=definite did not describe a sexual assault, 7=definitely described a sexual assault), and whether the target was to blame for the outcome of the scenario (0=strongly disagree, 10=strongly agree).*

**Results Study 4 Without Collapsing Across Condition**

Ratings of perceived coercion, *F*(3, 591) = 385.46, *p* < .001, *η^2^_partial_=*.66, consent, *F*(3, 591) = 337.40, *p <* .001, *η^2^_partial_=*.63, sexual assault, *F*(3, 591) = 487.14, *p* < .001, *η^2^_partial_=*.71, and victim blaming, *F*(3, 591) = 168.09, *p* < .001, *η^2^_partial_=*.46, significantly differed across scenarios (Table S3, Figure S4). Consistent with the findings from Study 1, the scenario in which both women had 1 shot was seen as the least coercive and least likely a sexual assault, and most consensual compared to all other scenarios, but the target of the interaction was also seen as the most blameworthy for what happened compared to the targets in all other scenarios (*ps*<=.001). Additionally, consistent with Study 1, the scenario in which the target woman drank 15 shots and her partner drank 1 was seen as the most coercive and most likely a sexual assault, and least consensual compared to all the other scenarios, but also was attributed the least amount of blame (*ps<=*.001). Furthermore, consistent with Study 2, the scenarios in which both women consumed 15 shots was seen as less coercive and less likely an assault, and more consensual than the scenario in which the target woman had 1 shot and her partner 15 (*ps<*.001), but not more or less blameworthy for the outcome of her actions.

**Figure S4.**

*Mean ratings of coercion, consent, sexual assault and victim blaming across scenarios with lesbian women in Study 4.*

***Note.*** *The x-axis captures the dependent variables and the y-axis the participant ratings for each DV. Each bar reflects the mean ratings for each DV per scenario described.* *Higher scores reflect greater endorsement that scenarios were coercive (1=completely disagree, 7=completely agree), consensual (1=definitely did not give consent, 7=definitely gave consent), and described a sexual assault (1=definite did not describe a sexual assault, 7=definitely described a sexual assault), and whether the target was to blame for the outcome of the scenario (0=strongly disagree, 10=strongly agree).*
